# Assessment of metal artifacts from titanium wrist prostheses: photon-counting *versus* energy-integrating detector CT

**DOI:** 10.1186/s41747-025-00587-w

**Published:** 2025-05-01

**Authors:** Nina Kämmerling, Simon Farnebo, Mårten Sandstedt, Ronald Booij, Anders Persson, Erik Tesselaar

**Affiliations:** 1 Department of Radiology, Department of Health, Medicine and Caring Sciences, Linköping University, Linköping, Sweden; 2 Center for Medical Image Science and Visualization (CMIV), Linköping University, Linköping, Sweden; 3 Department of Hand and Plastic Surgery, Department of Biomedical and Clinical Sciences, Linköping University, Linköping, Sweden; 4 Department of Radiology & Nuclear Medicine, Erasmus Medical Center, Rotterdam, The Netherlands; 5 Department of Medical Radiation Physics, Department of Health, Medicine and Caring Sciences, Linköping University, Linköping, Sweden

**Keywords:** Arthroplasty, Artifacts, Titanium, Tomography (x-ray computed), Wrist joint

## Abstract

**Background:**

We compared photon-counting detector computed tomography (PCD-CT) polyenergetic images, PCD-CT virtual monoenergetic images (VMI), and energy-integrating detector computed tomography (EID-CT) polyenergetic images regarding bone visualization and metal artifacts in patients with titanium wrist prostheses.

**Methods:**

After ethical approval, 15 patients were examined with PCD-CT and EID-CT. Polyenergetic images were reconstructed, as well as 130-keV VMI for PCD-CT. Five radiologists evaluated bone visualization, interpretability at metal-bone interface and metal artifacts using a 7-point ordinal scale. Streak artifacts and artifacts at the bone-metal interface were quantitatively assessed. Differences between image setups were analyzed using Friedman test and one-way ANOVA with *post hoc* tests.

**Results:**

Bone visualization was superior in PCD-CT polyenergetic images (median rating 6, range 3–7) compared with VMI (5, 3–7; *p* < 0.001) and EID-CT (5, 3–7; *p* = 0.018). Streak artifacts were more pronounced with PCD-CT polyenergetic images (4, 3–6) compared with EID-CT (5, 4–6; *p* = 0.003) and PCD-CT VMI (5, 3–7; *p* = 0.002), with quantitative results showing least streak artifacts in PCD-CT VMI, followed by EID-CT and PCD-CT polyenergetic images (50 ± 7%, 70 ± 6%, and 79 ± 5%, respectively; *p* < 0.001). Interpretability at bone-metal interface was better with PCD-CT polyenergetic images (5, 2–7; *p* = 0.045) and EID-CT (5, 3–6; *p* = 0.018) compared with PCD-CT VMI (4, 2–6), without quantitative differences.

**Conclusion:**

Streak artifacts from titanium wrist prostheses were reduced using 130-keV PCD-CT VMI, while bone visualization was highest using PCD-CT polyenergetic images.

**Relevance statement:**

In patients with wrist implants, photon-counting detector CT allows for effective metal artifact reduction using virtual monoenergetic images and improved bone visualization using polyenergetic images. As polyenergetic images and VMI have different advantages, access to both image setups may benefit diagnostic evaluation.

**Key Points:**

- Virtual monoenergetic images (VMI) presented a substantial reduction of metal streak artifacts.
- Polyenergetic images exhibited better image quality for bone imaging compared with VMI.
- A combination of image reconstructions should be preferred depending on the diagnostic task.

**Graphical Abstract:**

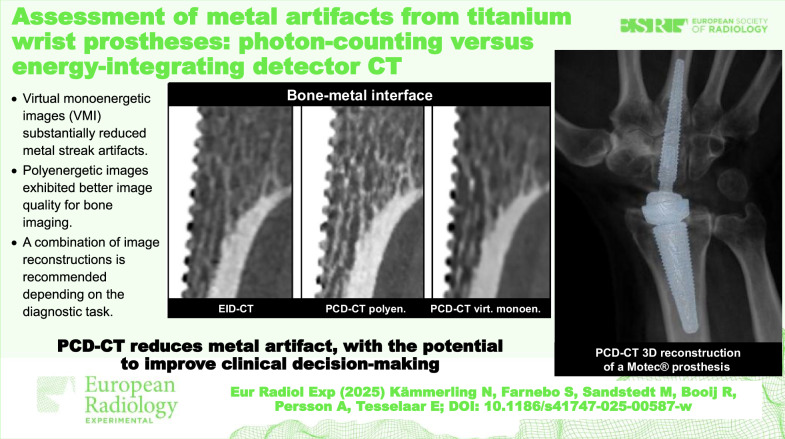

## Background

Complications of wrist implants, such as failure of osseointegration with implant loosening and osteolysis, are common causes of implant revision [1, 2]. Computed tomography (CT) can be used to diagnose these complications through analysis of the bone microstructure near the implant and at the interface between bone and metal. However, artifacts from the metal implant itself can cause degraded image quality. Typically, artifacts present as dark and bright streaks originating from the metal, or as a dark halo at the interface between metal and surrounding tissue. The causes for these effects are complex combinations of physical phenomena, such as beam hardening, photon scattering and starvation, and technical aspects of image acquisition and reconstruction, such as partial volume and edge effects [3–6]. The severity of the artifacts depends on the size and orientation of the implant, but also on its metallic composition [7].

Metal implants are commonly made of stainless steel (primarily composed of iron with atomic number 26), cobalt-chromium (atomic numbers 27 and 24, respectively), or titanium (atomic number 22). Larger metal implants and/or implants with a high atomic number, such as steel or cobalt-chromium, give rise primarily to artifacts caused by photon starvation. In this phenomenon, the high absorption of x-ray photons in the metal leads to an insufficient number of photons reaching the detector, which causes severe streaks. Titanium, with its lower atomic number, mainly causes beam-hardening artifacts. As the polychromatic x-ray beam passes the metal, the low-energy photons are absorbed to a larger extent, making the resulting beam consist of a higher proportion of high-energy photons. This energy shift is different in different projections, leading to inadequate data acquisition and thereby streak artifacts [5, 8–10].

There are different methods to reduce metal artifacts. One way is to increase tube current, whereby more photons contribute to the image. Increasing tube voltage leads to photons with higher energy, which results in better penetration and thus more photons reaching the detector. However, these methods have the disadvantage of a higher radiation exposure [10]. Tin prefiltration can be used to narrow the x-ray spectrum by removing low-energy photons, resulting in a higher proportion of photons contributing to the image, but this reduction is not always sufficient [8, 11]. Different metal artifact reduction software methods are available, but they are vendor-specific, and their effectiveness depends on the actual implant and anatomy of interest. These methods are primarily developed to compensate for photon starvation artifacts. The software itself can also introduce secondary artifacts, such as edge effects that can mimic implant loosening [4, 5, 12–14]. By using dual-energy CT, monoenergetic images can be generated, which, at high energy levels, are less susceptible to metal artifacts caused by beam hardening [10, 15, 16]. Despite several different techniques available to reduce metal artifacts, there is still no general solution available.

Over the last few years, photon-counting detector CT (PCD-CT) was introduced in clinical practice and has been shown to improve image quality in musculoskeletal imaging by a superior visualization of bone microstructure and fracture imaging [17–22]. As there is no need for septa between the detector elements, the radiation dose efficiency is higher, and pixels can be smaller, leading to superior spatial resolution. Electronic noise is absent due to an energy threshold set above the level of the quanta produced by electronics. Moreover, all x-ray photons exceeding this threshold are counted with equal weight, resulting in a higher image contrast. In addition to the polyenergetic images, routinely acquired with PCD-CT, virtual monoenergetic images (VMI) can be reconstructed by using the possibility of energy binning [6, 23, 24]. The energy spectrum consists of only one energy level in VMI, which effectively eliminates beam-hardening artifacts [5].

Several previous studies have shown that PCD-CT can reduce metal artifacts, and a few studies have evaluated bone imaging near implants and at the interface between bone and metal, which is the most important area for the detection of complications or implant failure [8, 25–28]. However, to our knowledge, no studies have been conducted specifically in patients with titanium implants. In CT imaging of titanium implants, the major cause of the metal artifacts is supposed to be beam hardening, known to be effectively suppressed by VMI. However, as wrist implants are relatively small, the effect of tin prefiltration and the inherent technical advantages in PCD-CT might be enough to reduce the artifacts in the strive to get as good bone-metal visibility as possible, especially as the artifact reduction by VMI comes at the expense of reduced spatial resolution.

The aim of this study was therefore to assess image quality and severity of metal artifacts in polyenergetic images and VMI of the wrist obtained with PCD-CT, compared with EID-CT images, in patients with titanium wrist prostheses.

## Methods

### Patients

The study was approved by the Swedish Ethical Review Authority (Dnr. 2021-02034).

**Fig. 1:**
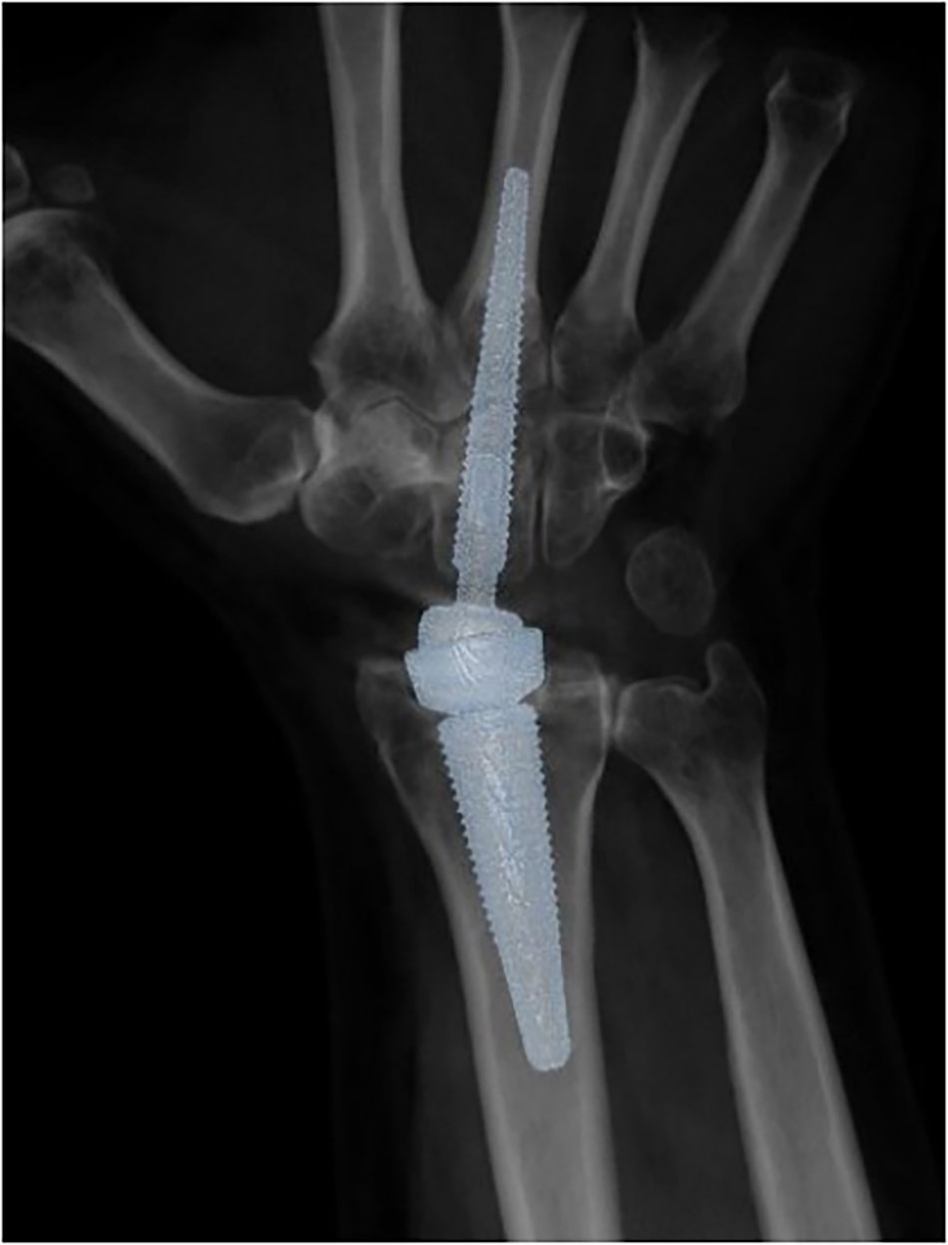
Three-dimensional reconstruction of a Motec® Wrist Joint Prosthesis acquired using photon-counting detector computed tomography (polyenergetic reconstruction)

Fifteen individuals (9 men and 6 women, mean age 67 years, range 54–78 years) eligible for surgery with total wrist arthroplasty at the Department of Hand and Plastic Surgery at Linköping University Hospital were recruited to participate in the study. Inclusion criteria were: total wrist arthroplasty using Motec® Wrist Joint Prosthesis (Motec, Swemac Innovation AB); being adult and non-pregnant; ability to participate; and provision of oral and written informed consent. The patients were examined with both EID-CT and PCD-CT the day after surgery. The Motec® Wrist Joint Prosthesis consists of a “ball-and-socket” prosthesis fixated in bone with one distal and one proximal threaded stem. The stems consist of titanium alloy (Fig. 1).

### Imaging protocol

An EID-CT scanner (SOMATOM Force, Siemens Healthineers) and a PCD-CT scanner (NAEOTOM Alpha, Siemens Healthineers) were used. For EID-CT, a clinical protocol with radiation dose determined by automatic tube current modulation (CARE Dose4D) was used (volume CT dose index (CTDI_vol_) 7.2 ± 0.1 mGy, mean ± standard deviation), and the PCD-CT dose was matched (CTDI_vol_ 7.4 ± 0.2 mGy). For both systems, the ultra-high-resolution mode was used. Axial images were reconstructed using the thinnest possible slice thickness on each system, *i.e*., 0.4 mm for EID-CT, 0.2 mm for PCD-CT polyenergetic images, and 0.4 mm for PCD-CT VMI reconstructed at 130 keV. Reconstruction kernels from our clinical protocols optimized for bone imaging (Ur73 for EID-CT and Br89 for PCD-CT polyenergetic images), as well as the sharpest kernel possible for PCD-CT VMI (Br76), were used. A detailed overview of acquisition and reconstruction parameters is given in Table 1.

**Table 1:** Acquisition and reconstruction parameters

Acquisition and reconstruction parameters	Energy-integrating detector CT	Photon-counting detector CT	
Scanner platform	Siemens SOMATOM Force	Siemens NAEOTOM Alpha	
Tube voltage/prefiltration (kVp)	Sn150	Sn140	
CTDI_vol32_ (mGy, mean ± standard deviation)	7.2 ± 0.1	7.4 ± 0.2	
Scan mode	Ultra-high resolution	Quantum HD	
Collimation	0.6 × 32 (total 19.2 mm)	0.2 × 120 (total 24 mm)	
Pitch	0.55	0.8	
Rotation time (s)	1.0	1.0	
Field-of-view (mm)	Patient-dependent according to clinical standard, ranging from 120 to 183 mm		
Spectral postprocessing	–	Polyenergetic	130-keV VMI
Image reconstruction kernel	Ur73	Br89	Br76
Iterative strength	ADMIRE 3	QIR3	
Slice thickness/increment (mm/mm)	0.4/0.2	0.2/0.1	0.4/0.2
Reconstruction matrix	512 × 512	1,024 × 1,024	

*CTDI*_*vol32*_ Volume computed tomography dose index with 32-cm phantom, *VMI* Virtual monoenergetic images

### Quantitative assessment of noise

A 5-mm^2^ circular region of interest was placed by one of the authors (N.K.) in muscle tissue in three consecutive slices proximal to the stem in radius. All the regions of interest were placed in similar positions. Noise was measured as standard deviation of the attenuation values in HU in muscle tissue.

### Quantitative assessment of streak artifacts

For each patient, a curved line was placed in muscle tissue 15 mm from the center of the implant. The length of the line was adjusted for each patient to ensure that only a minimal amount of other tissue, such as tendon or fat, was included. The attenuation values of the voxels along this line were measured.

**Fig. 2:**
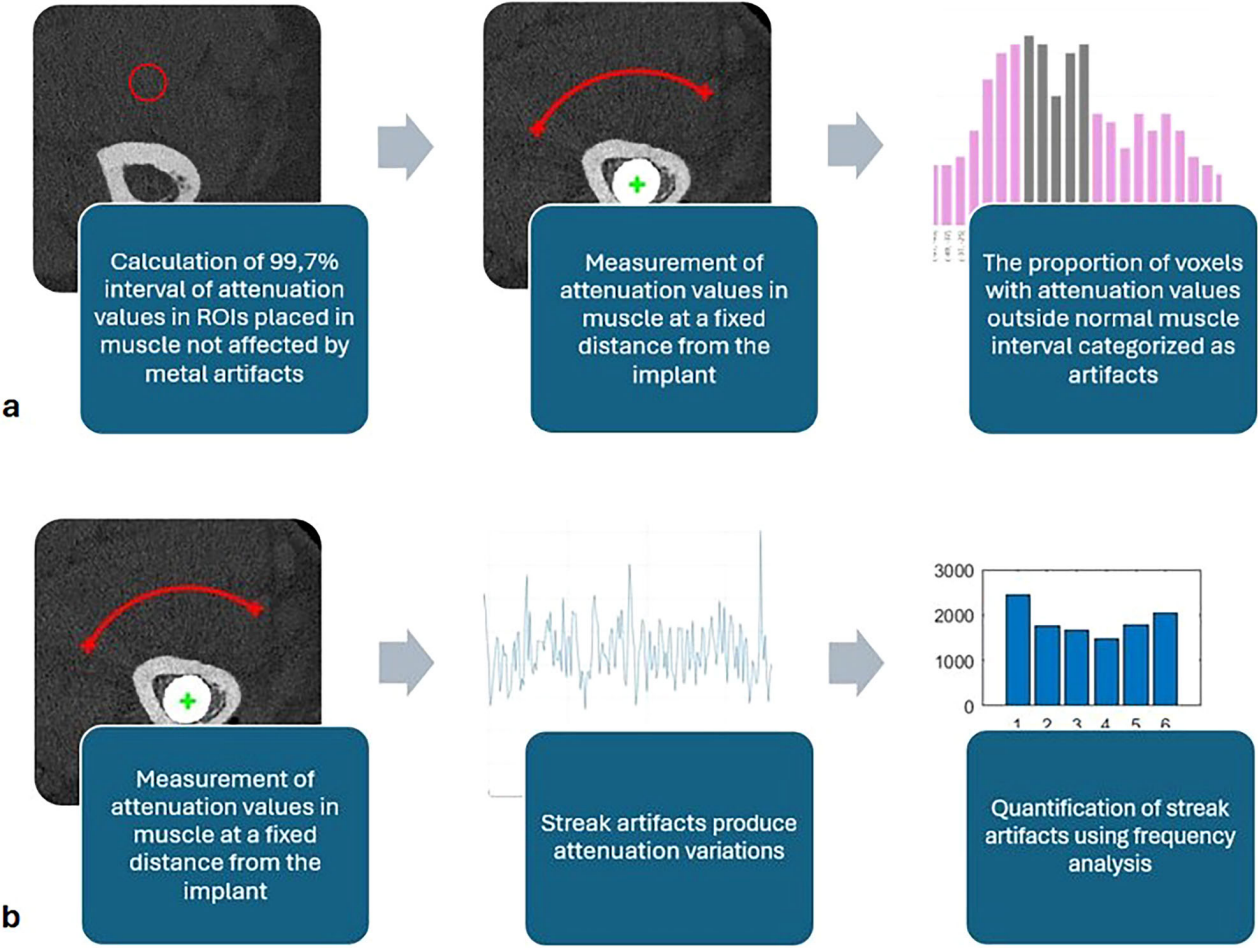
Description of the two different analysis methods used for the quantification of streak artifacts. **a** Quantification of proportion of artifacts in the image. **b** Quantification of frequency changes

Streak artifacts were assessed according to two methods: (1) calculation of the proportion of voxels containing artifacts, and (2) quantification of frequency changes (Fig. 2). Both methods were implemented using custom-made MATLAB scripts.

#### Method 1 (proportion of artifacts)

To calculate the proportion of artifacts, a method adapted from Do et al was used [29]. To define the expected range of attenuation values in HU for normal muscle tissue, we calculated mean and standard deviation within regions of interest in three consecutive slices of artifact-free muscle tissue proximal to the radial stem. The interval for normal muscle attenuation values was determined as the mean ± 3 standard deviations, which should encompass 99.7% of normal muscle tissue, assuming a normal distribution of attenuation values. Any voxels containing attenuation values outside this interval were classified as metal-induced artifacts. For each image, the proportion of voxels with attenuation values that fell outside the expected interval (*i.e*., voxels categorized as artifacts) was calculated. A higher proportion indicated the presence of more streak artifacts.

#### Method 2 (quantification of frequency changes)

To further quantify streak artifacts, a frequency domain-based method was employed, adapted from previous studies [30]. This approach leverages the observation that metal artifacts introduce oscillations, such as alternating “bright-dark” streaks, which are non-present in artifact-free images. Instead of using a full circle as described in previous studies, we selected an arc that was confined to muscle tissue, avoiding other anatomical structures. The arc was placed at a fixed 15-mm distance from the implant, ensuring the analysis focused on tissue likely to be affected by metal artifacts. Pixels along the arc were extracted in a counterclockwise direction, creating a 1-dimensional signal representing attenuation values. This signal was transformed into a spectrum of spatial frequencies using the Fast Fourier Transform. To quantify the artifact oscillations, the magnitude of each Fourier coefficient was calculated. We determined that the frequency bins 1 to 64 provided the most reliable measure of streak artifacts while remaining insensitive to variations in image noise. These bins were summed and analyzed to provide a quantifiable measure of the artifact intensity for each image setup.

### Quantitative assessment of undershoot/halo artifacts

To assess the nonlinearity of the image at the interface between the metal implant and the surrounding bone, we utilized a custom MATLAB script that generates 360° radial lines from the center of the implant, extending into the adjacent tissue. The attenuation values along each radial line were sampled at 0.05-mm intervals. For each line, the median CT number in the metal implant (HU_metal_), the median CT number in the bone (HU_bone_), and the minimum CT number along the line (HU_min_) were calculated. The relative undershoot (%U) at the bone-metal interface was calculated as:

$$\% U=\frac{{{HU}}_{{\rm{bone}}}-{{HU}}_{\min }}{{{HU}}_{{\rm{metal}}}-{{HU}}_{{\rm{bone}}}}\times 100$$

To ensure consistency between the CT number scales of PCD-CT and EID-CT, all attenuation values were truncated to a range between -1,024 and 3,072 HU.

### Qualitative assessments

Five general radiologists with 3, 5, 7, 10, and 20 years of experience, all used to read images with various orthopedic implants, participated in the observer study. The image stacks were presented in randomized order, with axial reconstructions on the left monitor and multiplanar reconstruction mode on the right monitor. The radiologists were blinded to the origin of the images and the reconstruction method. They could adjust the multiplanar reconstructions and window settings and zoom and pan the images according to their preferences. They were asked to assess the images regarding bone imaging in general, bone trabecular visibility as a measure of bone detail visibility, image quality at the interface between bone and metal, and prevalence of streak artifacts. Ordinal scales 1–7 were used in the assessments. The assessments asked for are shown in Table 2.

**Table 2:** Assessments and scales used in reader study

Assessments	Scale options
What is your opinion of image quality in general regarding bone imaging?	1 = very poor; 2 = poor; 3 = moderate/acceptable; 4 = fair; 5 = good; 6 = very good; 7 = excellent
What is your opinion of the visibility of the trabecular architecture?	
What is your opinion of the interpretability of the bone at the interface between bone and metal?	
What is your opinion regarding the presence of streak artifacts at the level of the stem component in radius?	1 = very severe, 2 = severe; 3 = quite severe; 4 = moderate: 5 = mild; 6 = very mild; 7 = not present

### Statistical analysis

Results of the quantitative analyses are given as mean ± standard deviation, while ratings in the reader study are presented as median and range (min to max). The statistical calculations were done using dedicated software (IBM SPSS Statistics for Windows, Version 29.0.0.0, Armonk, NY, USA). The proportion of artifacts in muscle (measured as attenuation values categorized as non-muscle) and undershoot were analyzed using one-way analyses of variance (ANOVA) corrected for multiple comparisons using the Tukey *post hoc* test. Noise and streak artifacts did not show homogeneity of variance according to Levene’s test, which is why Welch’s test and the Games-Howell *post hoc* test were used. A *p*-value lower than 0.05 was considered statistically significant.

To compare the three imaging setups in the observer study, we used Friedman test, a non-parametric alternative for repeated-measures data. *Post hoc* pairwise comparisons were adjusted by the Bonferroni correction. In addition, to evaluate the clinical relevance of our findings, we dichotomized the ratings at a threshold of 5 (very poor, poor, moderate/acceptable or fair *versus* good, very good, or excellent) and compared the proportions of favorable ratings across imaging setups. For these repeated binary outcomes, we used Cochran’s Q test. Interrater reliability was determined using intraclass correlation coefficient (ICC), with a two-way random model for consistency, single rating (k = 5). Interpretation of ICC values was performed in accordance with Koo and Lee [31].

## Results

### Quantitative assessments

An overview of the results of the quantitative assessments is given in Table 3.

**Table 3:** Overview of quantitative assessments

	EID-CT	PCD-CT polyenergetic images	PCD-CT 130-keV VMI
Noise as standard deviation of attenuation values in muscle (HU)	62.1 ± 7.2	79.9 ± 8.9***	25.9 ± 3.2^§§§,ααα^
Streak artifacts as proportion of voxels containing indicating artifacts	70 ± 6%	79 ± 5%***	50 ± 7%^§§§,ααα^
Streak artifacts quantified using the frequency-domain method	988 ± 495	1,430 ± 436*	492 ± 180^§§,ααα^
Relative undershoot at bone-metal interface (%)	23 ± 21	34 ± 36	24 ± 18

Data are given as mean ± standard deviation. Significant differences between EID-CT and PCD-CT polyenergetic images are indicated with * (*p* < 0.05), ** (*p* < 0.01), *** (*p* < 0.001), between EID-CT and PCD-CT VMI with ^§^, and between PCD-CT polyenergetic images and VMI with ^α^

*EID-CT* Energy-integrating detector computed tomography, *PCD-CT* Photon-counting detector computed tomography, *VMI* Virtual monoenergetic images

Noise was lowest with PCD-CT VMI, followed by EID-CT and PCD-CT polyenergetic images (25.9 ± 3.2, 62.1 ± 7.2 and 79.9 ± 8.9, respectively, *p* < 0.001 between all imaging methods).

The proportion of attenuation values indicating artifacts (method 1) was lowest in PCD-CT VMI, followed by EID-CT and PCD-CT polyenergetic images (50 ± 7%, 70 ± 6% and 79 ± 5%, respectively, *p* < 0.001 between all imaging methods).

Streak artifacts quantified using the frequency-domain approach (method 2) showed similar results, with lowest values observed in PCD-CT VMI followed by EID-CT and PCD-CT polyenergetic images (492 ± 180, 988 ± 495 and 1,430 ± 436, respectively), and the differences were significant (PCD-CT VMI *versus* EID-CT *p* = 0.005; PCD-CT VMI *versus* PCD-CT polyenergetic images *p* < 0.001, EID-CT *versus* PCD-CT polyenergetic images *p* = 0.039).

No significant differences in undershoot were observed between the different imaging methods (24 ± 18% for PCD-CT VMI, 23 ± 21% for EID-CT and 34 ± 36% for PCD-CT polyenergetic images (PCD-CT VMI *versus* EID-CT *p* = 0.998; PCD-CT VMI *versus* PCD-CT polyenergetic images *p* = 0.540, EID-CT *versus* PCD-CT polyenergetic images *p* = 0.449)).

### Qualitative assessments

**Fig. 3:**
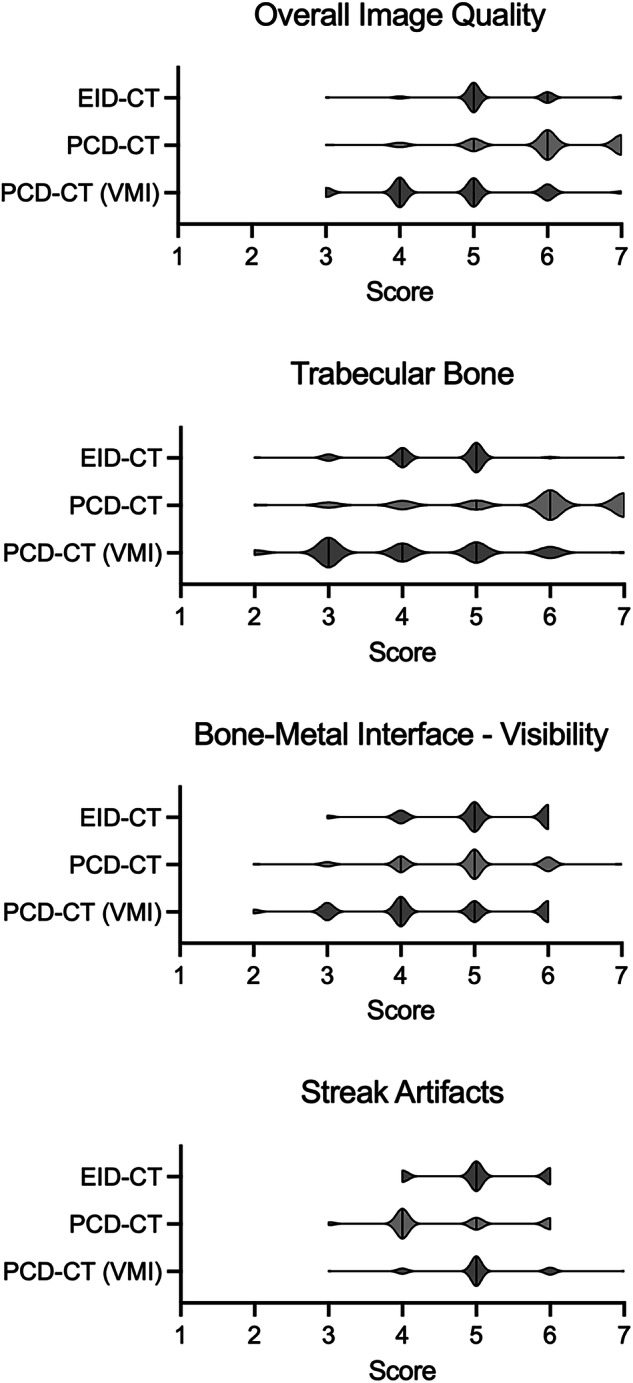
Observer ratings across imaging modalities. Violin plots depict ratings for overall image quality, visibility of trabecular bone structures, visibility of the bone-metal interface and streak artifacts. EID-CT, Energy-integrating detector computed tomography; PCD-CT, Photon-counting detector computed tomography; VMI, Virtual monoenergetic images

General image quality of bone was rated higher for PCD-CT polyenergetic images (median rating 6, range 3–7) than VMI (5, 3–7*; p* < 0.001) and EID-CT (5, 3–7; *p* = 0.018). There was a tendency of higher ratings with EID-CT compared with PCD-CT VMI, but the result was not significant (*p* = 0.201). The visibility of trabecular structures was rated higher with PCD-CT polyenergetic images (6, 2–7) compared with both EID-CT (5, 2–7; *p* = 0.001) and PCD-VMI (4, 2–7; *p* < 0.001). There was no significant difference between EID-CT and PCD-CT VMI (*p* = 0.273). The visualization of the interface between bone and metal was rated higher with PCD-CT polyenergetic images (5, 2–7; *p* = 0.045) and EID-CT (5, 3–6; *p* = 0.018) compared with PCD-CT VMI (4, 2–6), but there was no significant difference between PCD-CT polyenergetic images and EID-CT (*p* = 0.715). The observers considered streak artifacts to be more pronounced using PCD-CT polyenergetic images (4, 3–6) compared with both EID-CT (5, 4–6; *p* = 0.003) and PCD-CT VMI (5, 3–7; *p* = 0.002). No significant difference between EID-CT and PCD-CT VMI was shown (*p* = 0.855). The ratings by the observers are shown in Fig. 3.

When ratings were dichotomized at a threshold of 5, the proportions of favorable ratings varied significantly across imaging setups, consistent with the findings of the Friedman test (Table 4). Over 90% of images were rated as good quality for both EID-CT and PCD-CT polyenergetic images, compared to only 55% for PCD-CT VMI (*p* < 0.001). The visibility of trabecular bone structures was rated as good in 81% of PCD-CT polyenergetic images, significantly higher than the 55% and 39% observed for EID-CT and PCD-CT VMI, respectively (*p* < 0.001). The visualization of the bone-metal interface was rated as good in 77% of EID-CT images and in 68% for PCD-CT polyenergetic images, compared to 45% for PCD-CT VMI (*p* < 0.001 and *p* = 0.006, respectively). Lastly, streak artifacts were deemed non-interfering in 80% and 87% of EID-CT and PCD-CT VMI images, respectively, but in only 41% of PCD-CT polyenergetic images (*p* < 0.001).

**Table 4:** Percentages of readings rated as “good”, “very good”, or “excellent” for each of the four criteria

Criterion	EID-CT	PCD-CT polyenergetic images	PCD-CT 130-keV VMI
Overall image quality	93%	92%	55%^§§§,ααα^
Trabecular bone	55%	81%^***^	39%^ααα^
Bone-metal interface	77%	68%	45%^§§§,αα^
Streak artifacts	80%	41%^***^	87%^ααα^

Assessments per criterion: *n* = 75. Significant differences between EID-CT and PCD-CT polyenergetic images are indicated with ** (*p* < 0.01) or *** (*p* < 0.001), between EID-CT and PCD-CT VMI with ^§^, and between PCD-CT polyenergetic images and VMI with ^α^

*EID-CT* Energy-integrating detector computed tomography, *PCD-CT* Photon-counting detector computed tomography, *VMI* Virtual monoenergetic images

### Interobserver reliability

There was moderate interobserver reliability regarding image quality (ICC = 0.50) and the visualization of trabeculae (ICC = 0.53). The reliability was poor concerning the visualization at the bone-metal interface (ICC = 0.08) and the estimation of streak artifacts (ICC = 0.14).

## Discussion

In this study, we assessed image quality and severity of metal artifacts in PCD-CT images of the wrist in patients that had undergone total wrist replacements using a modular implant that has implanted titanium alloy screws in the radius and the third metacarpal bone. PCD-CT images were obtained using polyenergetic images and VMI and were compared with images obtained using EID-CT. The main finding was that no single reconstruction was superior in all aspects; PCD-CT VMI was found to reduce metal streak artifacts best, but polyenergetic images were superior in bone imaging and interpretability at the bone-metal interface.

In the observer study, radiologists considered streak artifacts, presented as dark and bright streaks originating from the metal, less pronounced using PCD-VMI or EID-CT compared with PCD-CT polyenergetic images. This was confirmed by the quantitative methods used to assess streak artifacts.

PCD-CT has inherent possibilities to reduce metal artifacts due to higher dose efficiency, smaller detector elements, and the absence of electronic noise. The finding that EID-CT performed better than PCD-CT polyenergetic images was therefore not expected. Possible explanations include the use of a sharper reconstruction kernel [32] or thinner slice thickness for the PCD-CT polyenergetic images. Also, beam-hardening and photon starvation artifacts may be more pronounced in PCD-CT polyenergetic images compared with EID-CT, due to the equal energy weighting of all photons in PCD-CT [33]. On the contrary, in VMI, the energy spectrum consists of one energy level only and a minimum of beam-hardening artifacts should be produced, and thus streak artifacts diminished. This is supported by the results in our study as streak artifacts were least presented in VMI images. It is also in agreement with the results in a cadaver study by Fukuda et al on a forearm specimen with an inserted titanium radial plate [33].

**Fig. 4:**
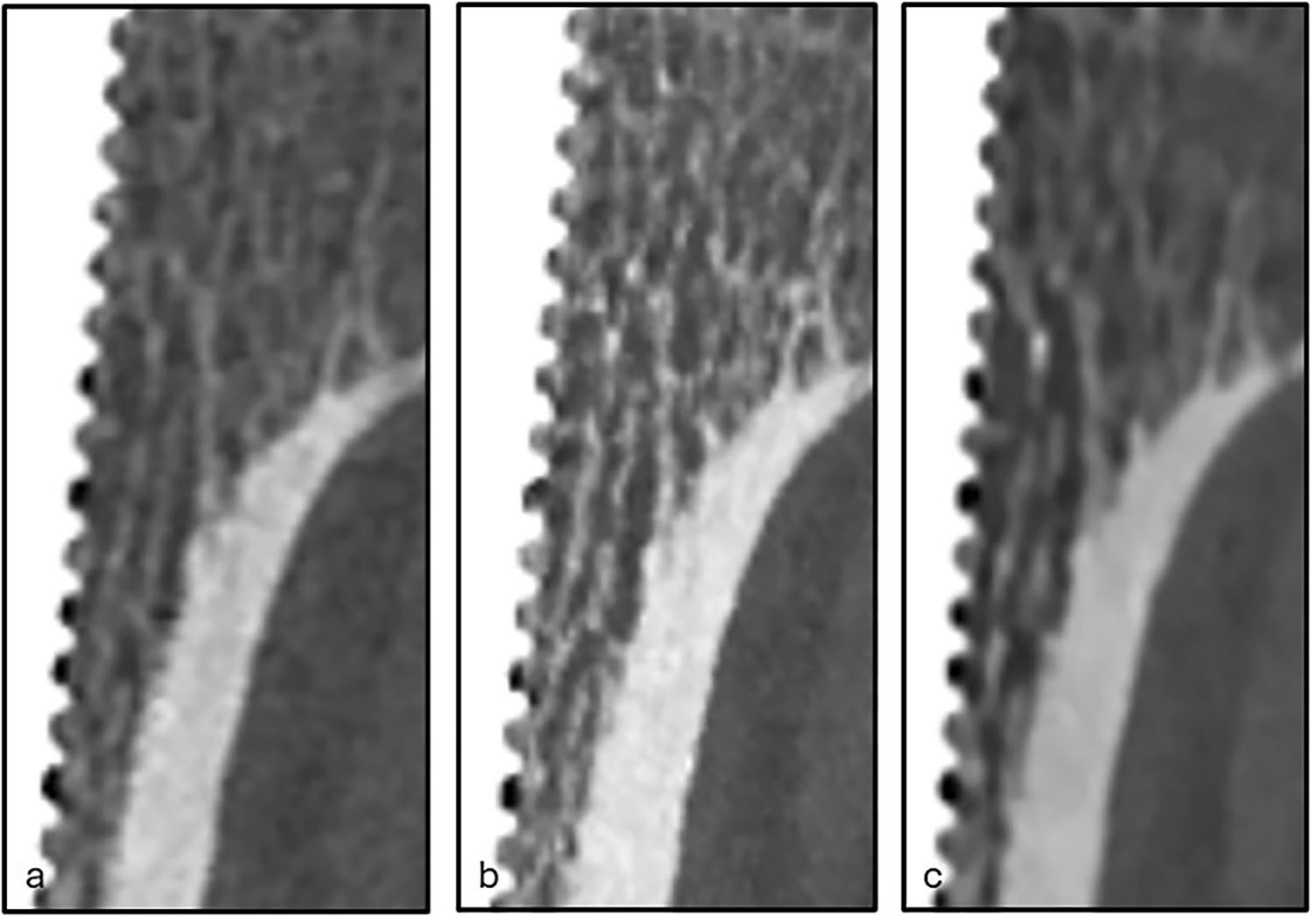
Coronal reconstructions of images obtained using EID-CT (**a**), PCD-CT polyenergetic images (**b**), and PCD-CT VMI (**c**), showing the interface between bone and metal implant. The visibility of trabecular structures was rated highest with PCD-CT polyenergetic images, followed by EID-CT and PCD-VMI. The interpretability of the interface was rated fair or above for all image setups. EID-CT, Energy-integrating detector computed tomography; PCD-CT, Photon-counting detector computed tomography; VMI, Virtual monoenergetic images

We found no significant difference between the three image reconstructions in the quantitative evaluation of artifacts at the bone-metal interface, which are measured as low-attenuation zones at the interface. However, the ratings by the five radiologists showed better interpretability at the interface with EID-CT and PCD-CT polyenergetic images compared to PCD-CT VMI. An example of each image reconstruction from one of the patients is shown in Fig. 4. Even though there was quantitatively measurable undershoot/low-attenuating zone at the bone-metal interface in all image setups, this might not have been clearly visible to the human eye, and other factors might have influenced the qualitative results in favor to PCD-CT polyenergetic images and EID-CT, such as image preferences or higher spatial resolution making it easier to evaluate the bone tissue at the interface. Another possible reason might be the different appearance of bone tissue in the indentations and the protrusions along the threaded stem, also related to spatial resolution.

We found that image quality in bone imaging in general and the visibility of the trabecular architecture were rated higher in PCD-CT polyenergetic images compared with both EID-CT and PCD-CT VMI, even though the noise level was highest in PCD-CT polyenergetic images. The superior visualization of bone is due to the inherent higher spatial resolution with PCD-CT compared with EID-CT, and the sharper kernel and the thinner slice thickness used for the PCD-CT polyenergetic images compared with both EID-CT and PCD-CT VMI [6, 19]. With the sharpest available kernel in VMI, Br76, the full potential of the improved spatial resolution, possible with PCD-CT polyenergetic images, is not used. The higher noise level received by sharper kernels and thin slices can be considered an acceptable trade-off for obtaining a higher spatial resolution.

A complementary analysis, in which observer ratings were dichotomized at a threshold of “good” or better, further supported our main findings. While both EID-CT and PCD-CT polyenergetic images achieved high proportions of favorable ratings for bone visualization and bone-metal interface, PCD-CT VMI performed better in the reduction of streak artifacts, again illustrating the trade-off between spatial resolution and artifact suppression.

In a clinical setting, there would ideally be a single imaging setup that perfectly combines artifact reduction and high spatial resolution. However, implant design, size and metal composition strongly influence the severity of artifacts. In our study on small titanium implants, tin prefiltration combined with the technical advantages in PCD-CT was not found to reduce metal streak artifact to a level of “good.” On the other hand, the superior reduction in streak artifacts by using VMI did not compensate for the reduced spatial resolution received regarding bone imaging compared with the polyenergetic images. Previous patient studies on small titanium implants used in the wrist are scarce, and studies on lower extremity implants of different metal compositions and designs have shown varied results. Woisetschläger et al [25] showed in their study on *ex vivo* hip prostheses superior visibility of the interface between bone and metal cup using PCD-CT polyenergetic images compared with EID-CT, but VMI was not evaluated. In a study by Marth et al [34], VMI was found to be preferred for metal artifact reduction and visualization of osseous healing compared with polychromatic images. Patzer et al [26] also found superior metal artifact reduction and bone assessability in VMI reconstructions, but additionally, they found that the results differed depending on the type of orthopedic implant/fixation. However, in the latter two studies, implants of different metal compositions were not analyzed separately, or the metal composition was not known. Hence, although there are improvements in artifact reduction and bone visualization using VMI and PCD-CT, a single acquisition and reconstruction protocol customized to all kinds of implants is still lacking.

Our study has several limitations. First, only 15 patients were included. Second, we only assessed metal artifacts from parts of the prosthesis that were made of titanium alloy. Even though the prosthesis contained cobalt-chrome-molybdenum parts, artifacts arising from those parts were not evaluated, as these parts were not directly surrounded by bone. Third, we did not compare different VMI energy levels (only the 130-keV energy level was studied). With other energy levels, the results concerning bone imaging as well as artifact reduction and interface visibility may have differed. Fourth, although the patients were positioned as consistently as possible, slight variations in the orientation of the implants between EID-CT and PCD-CT scans may have affected the metal artifacts. Fifth, interobserver reliability was moderate-to-low, especially for the bone-metal interface assessment. Differences in reader experience or subjective sensitivity to streak artifacts may lead to variation in qualitative ratings. A larger pool of readers, standardized scoring criteria, or consensus meetings could help reduce variability and strengthen confidence in the clinical applicability of our findings. Finally, we did not evaluate metal artifact reduction software. As metal artifact reduction software can produce secondary artifacts and cannot be combined with sharper bone kernels, the technique might be more suitable for visualization of soft tissue or in cases of more severe metal artifacts [10, 35].

In conclusion, in patients with total wrist arthroplasty with titanium prostheses, PCD-CT 130-keV VMI reduced metal streak artifacts better than polyenergetic PCD-CT and EID-CT images optimized for bone imaging. However, overall image quality, visualization of bone microstructure and interpretability at the bone-metal interface were considered better with PCD-CT polyenergetic images. These results indicate that with PCD-CT, a combination of polyenergetic and VMI reconstructions may benefit the diagnostic evaluation of patients with titanium wrist prostheses.

## Data Availability

The data that support the findings of this study are available from Region Östergötland, Sweden, upon reasonable request. Contact the corresponding author for data requests. Restrictions may apply due to licensing and/or regulatory reasons.

## Abbreviations

CT: Computed tomography
CTDI_vol_: Volume CT dose index
EID-CT: Energy-integrating detector CT
ICC: Intraclass correlation coefficient
PCD-CT: Photon-counting detector CT
VMI: Virtual monoenergetic images

## References

[1] Redfern JAI, Mehta N, Farnebo S et al (2024) Complication rates and modes of short and medium-term failure in Motec total wrist arthroplasty: an international cohort study. J Hand Surg Eur Vol 49:27–33. 10.1177/17531934231195689 37684024 10.1177/17531934231195689

[2] Boeckstyns MEH, Herzberg G (2024) Complications after total wrist arthroplasty. J Hand Surg Eur Vol 49:177–187. 10.1177/17531934231203297 38315136 10.1177/17531934231203297

[3] Barrett JF, Keat N (2004) Artifacts in CT: recognition and avoidance. Radiographics 24:1679–1691. 10.1148/rg.246045065 15537976 10.1148/rg.246045065

[4] Grandmougin A, Bakour O, Villani N et al (2020) Metal artifact reduction for small metal implants on CT: which image reconstruction algorithm performs better? Eur J Radiol 127:108970. 10.1016/j.ejrad.2020.108970 32289628 10.1016/j.ejrad.2020.108970

[5] Katsura M, Sato J, Akahane M, Kunimatsu A, Abe O (2018) Current and novel techniques for metal artifact reduction at CT: practical guide for radiologists. Radiographics 38:450–461. 10.1148/rg.2018170102 29528826 10.1148/rg.2018170102

[6] Selles M, van Osch JAC, Maas M, Boomsma MF, Wellenberg RHH (2024) Advances in metal artifact reduction in CT images: a review of traditional and novel metal artifact reduction techniques. Eur J Radiol 170:111276. 10.1016/j.ejrad.2023.111276 38142571 10.1016/j.ejrad.2023.111276

[7] Lee MJ, Kim S, Lee SA et al (2007) Overcoming artifacts from metallic orthopedic implants at high-field-strength MR imaging and multi-detector CT. Radiographics 27:791–803. 10.1148/rg.273065087 17495293 10.1148/rg.273065087

[8] Vellarackal AJ, Kaim AH (2021) Metal artefact reduction of different alloys with dual energy computed tomography (DECT). Sci Rep 11:2211. 10.1038/s41598-021-81600-1 33500471 10.1038/s41598-021-81600-1 PMC7838173

[9] Pettersson E, Back A, Thilander-Klang A (2021) Comparison of metal artefacts for different dual energy CT techniques. Radiat Prot Dosimetry 195:232–245. 10.1093/rpd/ncab105 34345904 10.1093/rpd/ncab105 PMC8507444

[10] Wellenberg RHH, Hakvoort ET, Slump CH, Boomsma MF, Maas M, Streekstra GJ (2018) Metal artifact reduction techniques in musculoskeletal CT-imaging. Eur J Radiol 107:60–69. 10.1016/j.ejrad.2018.08.010 30292274 10.1016/j.ejrad.2018.08.010

[11] Zhou W, Bartlett DJ, Diehn FE et al (2019) Reduction of metal artifacts and improvement in dose efficiency using photon-counting detector computed tomography and tin filtration. Invest Radiol 54:204–211. 10.1097/RLI.0000000000000535 30562270 10.1097/RLI.0000000000000535 PMC6434693

[12] Neroladaki A, Martin SP, Bagetakos I et al (2019) Metallic artifact reduction by evaluation of the additional value of iterative reconstruction algorithms in hip prosthesis computed tomography imaging. Medicine (Baltimore) 98:e14341. 10.1097/MD.0000000000014341 30732160 10.1097/MD.0000000000014341 PMC6380676

[13] Greffier J, Larbi A, Frandon J, Daviau PA, Beregi JP, Pereira F (2019) Influence of iterative reconstruction and dose levels on metallic artifact reduction: a phantom study within four CT systems. Diagn Interv Imaging 100:269–277. 10.1016/j.diii.2018.12.007 30709793 10.1016/j.diii.2018.12.007

[14] Layer YC, Mesropyan N, Kupczyk PA et al (2023) Combining iterative metal artifact reduction and virtual monoenergetic images severely reduces hip prosthesis-associated artifacts in photon-counting detector CT. Sci Rep 13:8955. 10.1038/s41598-023-35989-6 37268675 10.1038/s41598-023-35989-6 PMC10238372

[15] Bamberg F, Dierks A, Nikolaou K, Reiser MF, Becker CR, Johnson TR (2011) Metal artifact reduction by dual energy computed tomography using monoenergetic extrapolation. Eur Radiol 21:1424–1429. 10.1007/s00330-011-2062-1 21249370 10.1007/s00330-011-2062-1

[16] Wellenberg RH, Boomsma MF, van Osch JA et al (2017) Quantifying metal artefact reduction using virtual monochromatic dual-layer detector spectral CT imaging in unilateral and bilateral total hip prostheses. Eur J Radiol 88:61–70. 10.1016/j.ejrad.2017.01.002 28189210 10.1016/j.ejrad.2017.01.002

[17] Grunz JP, Huflage H, Heidenreich JF et al (2021) Image quality assessment for clinical cadmium telluride-based photon-counting computed tomography detector in cadaveric wrist imaging. Invest Radiol 56:785–790. 10.1097/RLI.0000000000000789 33882030 10.1097/RLI.0000000000000789

[18] Bette SJ, Braun FM, Haerting M et al (2022) Visualization of bone details in a novel photon-counting dual-source CT scanner-comparison with energy-integrating CT. Eur Radiol 32:2930–2936. 10.1007/s00330-021-08441-4 34936011 10.1007/s00330-021-08441-4 PMC9038873

[19] Kammerling N, Sandstedt M, Farnebo S, Persson A, Tesselaar E (2022) Assessment of image quality in photon-counting detector computed tomography of the wrist—an ex vivo study. Eur J Radiol 154:110442. 10.1016/j.ejrad.2022.110442 35849959 10.1016/j.ejrad.2022.110442

[20] Booij R, Kammerling NF, Oei EHG, Persson A, Tesselaar E (2023) Assessment of visibility of bone structures in the wrist using normal and half of the radiation dose with photon-counting detector CT. Eur J Radiol 159:110662. 10.1016/j.ejrad.2022.110662 36565594 10.1016/j.ejrad.2022.110662

[21] Rajendran K, Baffour F, Powell G et al (2023) Improved visualization of the wrist at lower radiation dose with photon-counting-detector CT. Skeletal Radiol 52:23–29. 10.1007/s00256-022-04117-2 35831718 10.1007/s00256-022-04117-2

[22] Kammerling N, Tesselaar E, Booij R, Fornander L, Persson A, Farnebo S (2024) A comparative study of image quality and diagnostic confidence in diagnosis and follow-up of scaphoid fractures using photon-counting detector CT and energy-integrating detector CT. Eur J Radiol 173:111383. 10.1016/j.ejrad.2024.111383 38377892 10.1016/j.ejrad.2024.111383

[23] Leng S, Bruesewitz M, Tao S et al (2019) Photon-counting detector CT: system design and clinical applications of an emerging technology. Radiographics 39:729–743. 10.1148/rg.2019180115 31059394 10.1148/rg.2019180115 PMC6542627

[24] Flohr T, Schmidt B (2023) Technical basics and clinical benefits of photon-counting CT. Invest Radiol 58:441–450. 10.1097/RLI.0000000000000980 37185302 10.1097/RLI.0000000000000980 PMC10259209

[25] Woisetschlager M, Booij R, Tesselaar E, Oei EHG, Schilcher J (2023) Improved visualization of the bone-implant interface and osseointegration in ex vivo acetabular cup implants using photon-counting detector CT. Eur Radiol Exp 7:19. 10.1186/s41747-023-00335-y 37121937 10.1186/s41747-023-00335-y PMC10149426

[26] Patzer TS, Grunz JP, Huflage H et al (2024) Ultra-high resolution photon-counting CT with tin prefiltration for bone-metal interface visualization. Eur J Radiol 170:111209. 10.1016/j.ejrad.2023.111209 37992609 10.1016/j.ejrad.2023.111209

[27] Schreck J, Laukamp KR, Niehoff JH et al (2023) Metal artifact reduction in patients with total hip replacements: evaluation of clinical photon counting CT using virtual monoenergetic images. Eur Radiol 33:9286–9295. 10.1007/s00330-023-09879-4 37436505 10.1007/s00330-023-09879-4 PMC10667386

[28] Bjorkman AS, Malusek A, Gauffin H, Persson A, Koskinen SK (2023) Spectral photon-counting CT: image quality evaluation using a metal-containing bovine bone specimen. Eur J Radiol 168:111110. 10.1016/j.ejrad.2023.111110 37788519 10.1016/j.ejrad.2023.111110

[29] Do TD, Sawall S, Heinze S et al (2020) A semi-automated quantitative comparison of metal artifact reduction in photon-counting computed tomography by energy-selective thresholding. Sci Rep 10:21099. 10.1038/s41598-020-77904-3 33273590 10.1038/s41598-020-77904-3 PMC7713179

[30] Grosse Hokamp N, Eck B, Siedek F et al (2020) Quantification of metal artifacts in computed tomography: methodological considerations. Quant Imaging Med Surg 10:1033–1044. 10.21037/qims.2020.04.03 32489927 10.21037/qims.2020.04.03 PMC7242301

[31] Koo TK, Li MY (2016) A guideline of selecting and reporting intraclass correlation coefficients for reliability research. J Chiropr Med 15:155–163. 10.1016/j.jcm.2016.02.012 27330520 10.1016/j.jcm.2016.02.012 PMC4913118

[32] Konst B, Ohlsson L, Henriksson L, Sandstedt M, Persson A, Ebbers T (2024) Optimization of photon counting CT for cardiac imaging in patients with left ventricular assist devices: an in-depth assessment of metal artifacts. J Appl Clin Med Phys 25:e14386. 10.1002/acm2.14386 38739330 10.1002/acm2.14386 PMC11244676

[33] Fukuda T, Yonenaga T, Akao R et al (2024) Comparison of bone evaluation and metal artifact between photon-counting CT and five energy-integrating-detector CT under standardized conditions using cadaveric forearms. Diagnostics (Basel) 14:350. 10.3390/diagnostics14040350 38396389 10.3390/diagnostics14040350 PMC10888094

[34] Marth AA, Goller SS, Kajdi GW, Marcus RP, Sutter R (2024) Photon-counting detector CT: clinical utility of virtual monoenergetic imaging combined with tin prefiltration to reduce metal artifacts in the postoperative ankle. Invest Radiol 59:545–553. 10.1097/RLI.0000000000001058 38214560 10.1097/RLI.0000000000001058

[35] Midthun P, Kirkhus E, Osteras BH, Hoiness PR, England A, Johansen S (2023) Metal artifact reduction on musculoskeletal CT: a phantom and clinical study. Eur Radiol Exp 7:46. 10.1186/s41747-023-00354-9 37524994 10.1186/s41747-023-00354-9 PMC10390408

